# Multiple treatment comparisons in epilepsy monotherapy trials

**DOI:** 10.1186/1745-6215-8-34

**Published:** 2007-11-05

**Authors:** Catrin Tudur Smith, Anthony G Marson, David W Chadwick, Paula R Williamson1

**Affiliations:** 1 Centre for Medical Statististcs and Health Evaluation, University of Liverpool, Liverpool, UK; 2 Division of Neuroscience, University of Liverpool, Liverpool, UK

## Abstract

**Background:**

The choice of antiepileptic drug for an individual should be based upon the highest quality evidence regarding potential benefits and harms of the available treatments. Systematic reviews and meta-analysis of randomised controlled trials should be a major source of evidence supporting this decision making process. We summarise all available individual patient data evidence from randomised controlled trials that compared at least two out of eight antiepileptic drugs given as monotherapy.

**Methods:**

Multiple treatment comparisons from epilepsy monotherapy trials were synthesized in a single stratified Cox regression model adjusted for treatment by epilepsy type interactions and making use of direct and indirect evidence. Primary outcomes were time to treatment failure and time to 12 month remission from seizures. A secondary outcome was time to first seizure.

**Results:**

Individual patient data for 6418 patients from 20 randomised trials comparing eight antiepileptic drugs were synthesized. For partial onset seizures (4628 (72%) patients), lamotrigine, carbamazepine and oxcarbazepine provide the best combination of seizure control and treatment failure. Lamotrigine is clinically superior to all other drugs for treatment failure but estimates suggest a disadvantage compared to carbamazepine for time to 12 month remission [Hazard Ratio (95% Confidence Interval) = 0.87(0.73 to 1.04)] and time to first seizure [1.29(1.13 to 1.48)]. Phenobarbitone may delay time to first seizure [0.77(0.61 to 0.96)] but at the expense of increased treatment failure [1.60(1.22 to 2.10)]. For generalized onset tonic clonic seizures (1790 (28%) patients) estimates suggest valproate or phenytoin may provide the best combination of seizure control and treatment failure but some uncertainty remains about the relative effectiveness of other drugs.

**Conclusion:**

For patients with partial onset seizures, results favour carbamazepine, oxcarbazepine and lamotrigine. For generalized onset tonic clonic seizures, results favour valproate and phenytoin.

## Background

Epilepsy is a common neurological condition with a prevalence of around 0.5% and a life time incidence of around 3% [1]. The majority of people with epilepsy are treated with a single (monotherapy) antiepileptic drug (AED), and 60–70% of patients will enter a remission from seizures soon after starting treatment, often on a first AED [2,3]. Current guidelines recommend valproate (VPA) as a treatment of first choice for patients with generalized onset seizures whilst carbamazepine (CBZ) is recommended as the first line treatment for patients with partial onset seizures [4,5]. However, with several AEDs to choose between and a number of new drugs licensed and introduced over the last decade and a half [6,7], it is essential to evaluate the effectiveness of these drugs against each other in the best possible way. The choice of AED for an individual should be based upon the highest quality evidence regarding potential benefits and harms of the available treatments. Systematic reviews and meta-analysis of randomised controlled trials should be a major source of evidence supporting this decision making process.

Eight separate Cochrane systematic reviews with meta-analysis have been prepared, or are in preparation, in which the following AED comparisons are made: CBZ against VPA [8], phenytoin (PHT) against VPA [9], CBZ against PHT [10], PHT against phenobarbitone (PB) [11], CBZ against PB [12], VPA against PB (review in preparation), oxcarbazepine (OXC) against PHT [13] and lamotrigine (LTG) against CBZ [14]. An individual patient data (IPD) approach using full original trial data sets rather than published or aggregate level data, has been used in these reviews, an approach that is regarded as the gold standard for meta-analysis [15]. Each Cochrane review provides the best available evidence about the comparative effects of each pair of drugs (pair-wise direct comparison). However, in isolation, each pair-wise comparison does not inform a choice among all these drugs and interpretation of the complete evidence base can be difficult for the physician or patient. Further difficulties arise with this current evidence base since direct evidence is not available for some pair-wise comparisons. For example, evidence directly comparing OXC with LTG or PB does not currently exist from a randomised controlled trial and, for OXC compared to PB, is unlikely to be examined in future trials because of changes in 'fashions' for treatment.

Meta-analyses of randomised controlled trials traditionally focus on the pair-wise comparison between two treatments, say A and B, with each included trial providing information for the direct comparison between A and B. For some treatment comparisons, randomised controlled trials may not exist therefore the treatment effect cannot be directly estimated. However, it is possible to estimate the indirect treatment effect of A versus B using evidence from trials comparing drug A with C, and trials comparing drug B with C. The key assumption for the indirect comparison is one of exchangeability of the treatment effect across all included trials such that the effect of A versus B would be expected to be similar if estimated within the trials that actually did compare A versus B or likewise within the trials that only compared B versus C. This assumption may be reasonable if the trial setting and clinical characteristics are similar or if any differences between trials would not be expected to modify the relative treatment effects. Although no formal statistical test exists to examine this assumption, careful consideration should be given for the reliability of this assumption.

Indirect comparisons can be valuable in situations particularly where direct comparisons either do not exist, comprise a limited amount of data, or are unlikely to ever be examined in future trials. Methods for estimating an indirect comparison using aggregate data have been discussed previously [16] and compared to direct evidence across 44 systematic reviews by Song et al [17]. They concluded that indirect comparisons usually (but not always) agree with the results of direct randomised trials with their validity dependent upon the internal validity and similarity of the included trials.

In this paper we describe a simultaneous analysis of IPD from randomised controlled trials included across the eight original Cochrane reviews along with subsequently obtained IPD from the largest ever randomised trial in epilepsy patients [6,7]. These randomised trials contribute direct and indirect evidence for multiple treatment comparisons of eight different AEDs that have a license for use as monotherapy in at least one country and will provide the best available overall summary of their comparative effects. This analysis will aid an evidence-based decision making process and simplify interpretation of the evidence for the physician and patient. The approach could be expanded to incorporate data for other AEDs once available, and the methods are relevant to many other areas of medicine where choices have to be made among a number of treatment options.

There is increasing recognition of a need to use multiple treatment comparisons with direct and indirect evidence to inform healthcare decisions [18] and it appears the logical next step following individual pair-wise meta-analyses. To our knowledge, this is the first such analysis based on IPD which is recognised to be the gold standard approach both in pair-wise meta-analyses and therefore, we believe, for multiple treatment comparison analyses.

## Methods

### IPD reviews

Each Cochrane systematic review of epilepsy monotherapy trials used identical protocol and review methodology. The search strategy included searching Medline 1966–2006 using the Cochrane Epilepsy Groups comprehensive search strategy, the Cochrane Controlled Trials Register and the Cochrane Epilepsy Group's register of randomised controlled trials. In each review, trials were included if they were double (patient and clinician), single (patient or clinician) or unblinded randomised controlled trials in which the two treatments of interest in the particular review were compared directly. Trial participants were children or adults with a new diagnosis of epilepsy, or a relapse following AED withdrawal, or who had failed on other therapies. Patients' seizures were classified as either partial onset (simple partial, complex partial, or secondarily generalising tonic-clonic seizures) or as generalised onset tonic-clonic seizures. Trials recruiting patients with other generalized seizure types (primarily absence and Myoclonic seizures) in the absence of tonic clonic seizures were excluded. All of the trials included in the review were undertaken after the publication of the 1981 International League Against Epilepsy (ILAE) classification of epileptic seizures [19], in which the distinction between partial onset and generalized onset seizures is made, hence patients could be split into partial onset and generalized onset seizure subgroups. However, many of the studies were initiated prior to the 1989 ILAE classification of epileptic syndromes [20]; hence individual epilepsy syndromes could not be investigated.

Three time-to-event outcomes were investigated, which are those recommended by the ILAE [20]. The primary outcomes were (i) time to treatment failure due to inadequate seizure control, intolerable adverse effects or a combination of both; a combined outcome reflecting both efficacy and tolerability and an outcome to which the individual makes a contribution, and (ii) time to 12 month remission from seizures, defined as the number of days between randomisation and end of a period of 12 months without seizures. A secondary outcome was time to first seizure after randomisation. Further details regarding specific review methodology are described in the relevant review publication.

### SANAD data

SANAD (A RCT Of Longer-Term Clinical Outcomes And Cost Effectiveness Of Standard And New Antiepileptic Drugs) was an unblinded randomized controlled trial recruiting patients between January 1999 and August 2004. Arm A [6] recruited patients for whom CBZ was considered to be standard treatment and they were randomized to CBZ, gabapentin (GBP), LTG, OXC or topiramate (TPM). Arm B [7] recruited patients for whom VPA was considered to be standard treatment and they were randomized to VPA, LTG, or TPM. Clinicians were asked to classify seizures and epilepsy syndromes by ILAE classifications as far as was possible, at least to differentiate between partial onset (focal) or generalised onset seizures. Primary outcomes were time to treatment failure, and time to 12 month remission. Secondary outcomes included time to first seizure. IPD are available for all participants.

### Simultaneous analysis of multiple treatment comparison

All analyses are by intention to treat as far as possible. As time to event outcomes are of interest, three Cox proportional hazards models are fitted to the data, one model for each outcome. To preserve the advantages of randomisation within each trial, the Cox model is stratified by trial including all patients from all treatment groups in all trials for which data are available [21]. The eight AEDs (CBZ, VPA, PHT, PB, OXC, GBP, TPM and LTG) of interest may be represented in the Cox proportional hazards model by seven dummy variables. Estimates of the hazard ratio and its standard error for each pair-wise comparison may be obtained from this model with appropriate recognition of the covariance between regression coefficients. With 8 AEDs, there are 28 possible pair-wise comparisons. For ease of presentation, we present hazard ratio estimates for each drug compared to the current standard (CBZ for partial and VPA for generalised) in this paper. However, estimates for all pair-wise comparisons were examined for conclusions and are displayed in Figure X [see additional file 1]. Further details regarding the model, model fit and calculation of hazard ratios and confidence intervals are given in Appendix 1 and discussed in more detail by Tudur Smith [22].

### Exchangeability

The assumption of exchangeability implies that the hazard ratio for each treatment comparison is similar across all covariate values i.e. there are no treatment by covariate interactions. This assumption is clinically unlikely for the epilepsy monotherapy comparisons since there are strong prior beliefs that some AEDs have different effects for generalised and partial onset seizures, i.e. there is a treatment by seizure type (partial versus generalized onset) interaction. For example, VPA is considered the treatment of choice for generalized seizures and epilepsy syndromes and CBZ the treatment of choice for partial seizures and epilepsy syndrome [23]. The availability of IPD for this particular example enables analyses that adjust for the effect of epilepsy type and interactions with treatments to improve the application of results to specific patient populations. This is achieved in the analysis by including terms to represent the main effects of treatment and epilepsy type and their interaction in the Cox model.

One further concern is that the individual trial data may not necessarily be contemporaneous. In particular, data for PB comes from trials that recruited patients between 1978 and 1987 whereas data for LTG comes from trials that recruited between 1989 and 2004. Both drugs have been compared against CBZ for which we have data across the period between 1978 and 2004. The assumption of exchangeability implies that the hazard ratio for PB versus CBZ, as estimated by the trials recruiting between 1978 and 1987, would also be expected if trials had compared PB and CBZ between 1989 and 2004. It is possible that improvements in clinicians' understanding of how to use CBZ could change over the time period between 1978 and 2004. For example, clinicians may be less tolerant of known adverse effects or may alter their pattern of dose escalation or maintenance dose. Such changes in practice could improve outcome over time for patients on CBZ which could invalidate the assumption of exchangeability. This hypothesis is explored by adding year of randomisation (as a categorical variable) for patients on CBZ to a Cox regression model with know prognostic factors (number seizures before randomisation and epilepsy type).

### Consistency

For each comparison where direct evidence is available, internal consistency of the direct evidence is assessed by visual inspection of confidence intervals for each trial hazard ratio, along with a chi-square test for heterogeneity and calculation of the I^2 ^statistic [24] for partial and generalised seizures separately. Consistency between the direct and combination of direct and indirect evidence is assessed by visual comparison of the hazard ratio estimates and 95% confidence intervals from each direct (seizure type specific) comparison with the corresponding combined result.

## Results

IPD are available for at least one outcome for 6418 patients with epilepsy type data across 20 randomised controlled trials [25-41]. There are 5817 patients [1552 (27%) generalised; 4265 (73%) partial] randomised within 17 trials available for the analysis of time to treatment failure, 4886 patients [1360 (28%) generalised; 3526 (72%) partial] from 14 trials for the analysis of time to 12 month remission and 5724 patients [1765 (31%) generalised; 3959 (69%) partial] from 19 trials for the analysis of time to first seizure. The summary of characteristics (Table 1) [see additional file 2] suggests clinical comparability across included trials and adds further support to the clinical justification for combining evidence from these multiple sources. A meaningful summary of titration schedules could not be created due to the inadequacy of data in trial publications.

**Figure 1:**
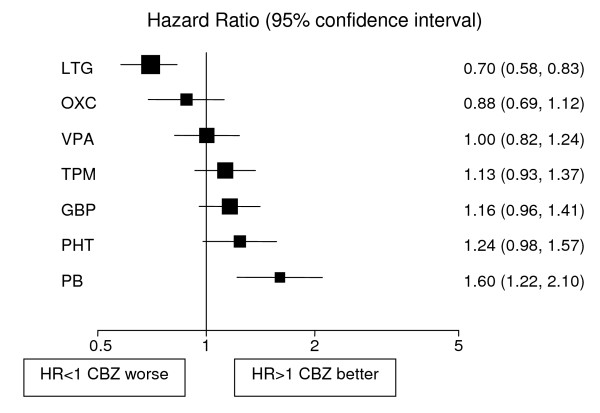
**Time to treatment failure for partial onset seizures (Hazard Ratio for each AED compared to standard CBZ)**. **CBZ**: Carbamazepine, **VPA**: Sodium Valproate, **PHT**: Phenytoin, **PB**: Phenobarbitone, **LTG**: Lamotrigine, **OXC**: Oxcarbazepine, **GBP**: Gabapentine, **TPM**: Topirimate

**Figure 2:**
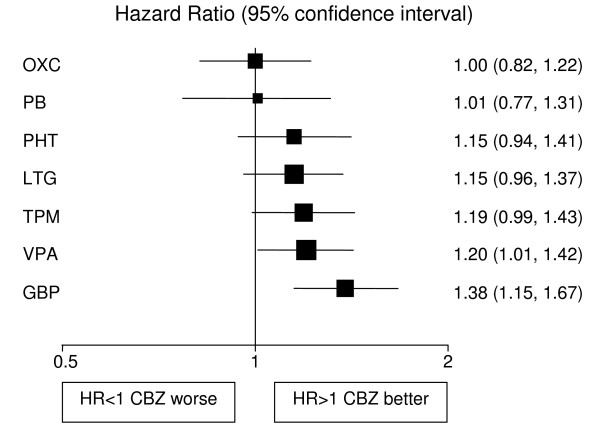
**Time to 12 month remission for partial onset seizures (Hazard Ratio for each AED compared to standard CBZ)**. **CBZ**: Carbamazepine, **VPA**: Sodium Valproate, **PHT**: Phenytoin, **PB**: Phenobarbitone, **LTG**: Lamotrigine, **OXC**: Oxcarbazepine, **GBP**: Gabapentine, **TPM**: Topirimate

**Figure 3:**
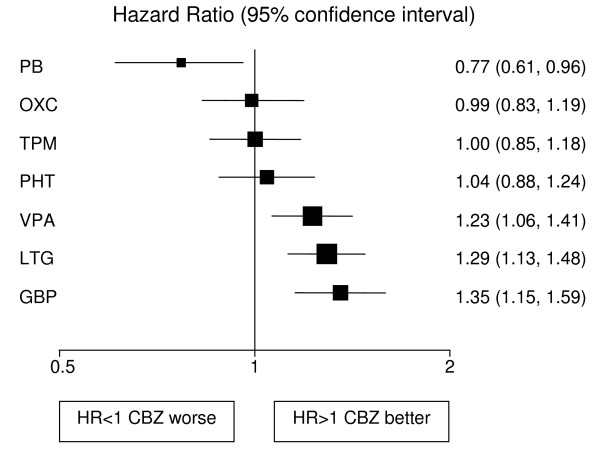
**Time to first seizure for partial onset seizures (Hazard Ratio for each AED compared to standard CBZ)**. **CBZ**: Carbamazepine, **VPA**: Sodium Valproate, **PHT**: Phenytoin, **PB**: Phenobarbitone, **LTG**: Lamotrigine, **OXC**: Oxcarbazepine, **GBP**: Gabapentine, **TPM**: Topirimate

**Figure 4:**
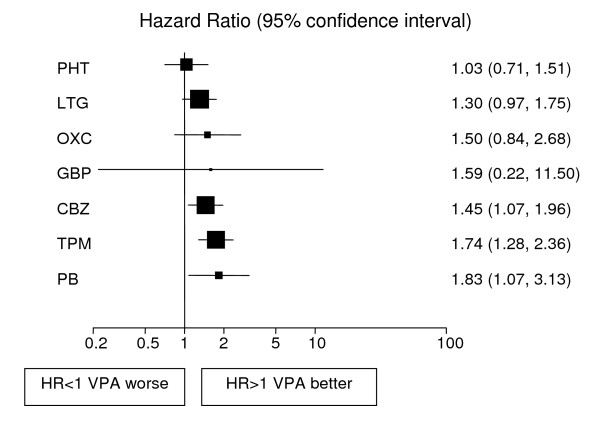
**Time to treatment failure for generalised onset seizures (Hazard Ratio for each AED compared to standard VPA)**. **CBZ**: Carbamazepine, **VPA**: Sodium Valproate, **PHT**: Phenytoin, **PB**: Phenobarbitone, **LTG**: Lamotrigine, **OXC**: Oxcarbazepine, **GBP**: Gabapentine, **TPM**: Topirimate

**Figure 5:**
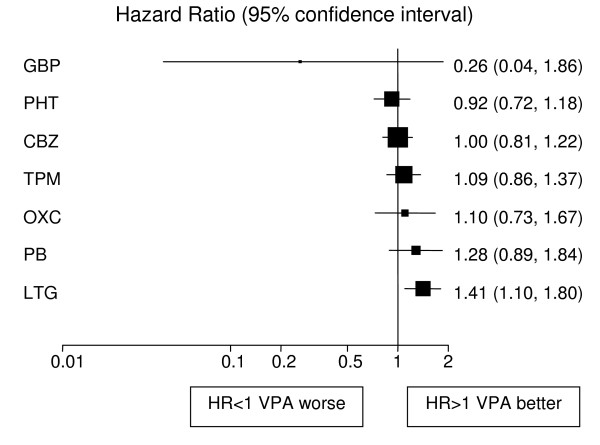
**Time to 12 month remission for generalised onset seizures (Hazard Ratio for each AED compared to standard VPA)**. CBZ: Carbamazepine, **VPA**: Sodium Valproate, **PHT**: Phenytoin, **PB**: Phenobarbitone, **LTG**: Lamotrigine, **OXC**: Oxcarbazepine, **GBP**: Gabapentine, **TPM**: Topirimate

**Figure 6:**
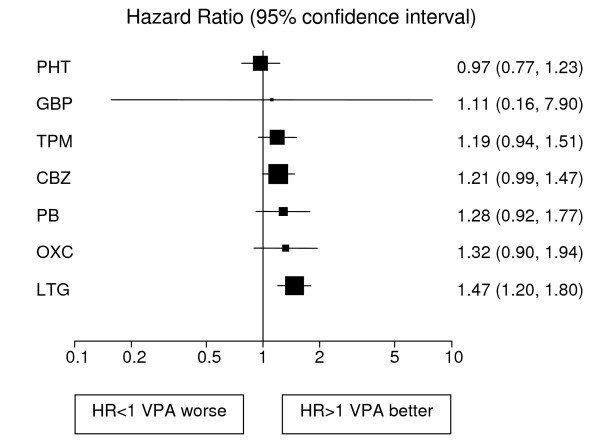
**Time to first seizure for generalised onset seizures (Hazard Ratio for each AED compared to standard VPA)**. **CBZ**: Carbamazepine, **VPA**: Sodium Valproate, **PHT**: Phenytoin, **PB**: Phenobarbitone, **LTG**: Lamotrigine, **OXC**: Oxcarbazepine, **GBP**: Gabapentine, **TPM**: Topirimate

Analyses adjusted for treatment and epilepsy type main effects and their interaction are displayed in Figures 1, 2, 3, 4, 5, 6 for each outcome based on the multiple treatment comparisons analysis of all relevant trials for which data are available. Each figure presents a hazard ratio and 95% confidence interval for every AED compared with the current standard (CBZ for partials and VPA for generalised) ordered by size of effect. Each box represents a point estimate of hazard ratio with the size of each box inversely proportional to the width of the 95% confidence interval for the relevant hazard ratio.

### Partial onset seizures

4628 (72%) patients were classified as having partial onset seizures, and results are presented in figures 1, 2, 3. For the first primary outcome time to treatment failure (Figure 1), LTG is the best drug (LTG is significantly better than all other AEDs apart from OXC), and PB is the worst drug. There is a non-significant trend to suggest that OXC may be better than CBZ but insufficient evidence to differentiate between CBZ and VPA. There are further non-significant trends to suggest that CBZ may be better than TPM, GBP and PHT with the lower limits of the 95% confidence interval limits not reaching clinical significance. For time to 12-month remission (Figure 2) CBZ is significantly better than GBP and VPA. There are non-significant trends showing benefit for CBZ over TPM, LTG and PHT with lower limits to the 95% confidence intervals close to 1.0. There is insufficient evidence to conclude whether there are differences between CBZ and OXC or PB with wide confidence intervals that include clinically important differences in both directions. For the secondary outcome, time to first seizure (Figure 3), CBZ is significantly better than VPA, LTG and GBP but there is insufficient evidence to conclude whether there are differences between CBZ and OXC, TPM or PHT with wide confidence intervals that contain clinically important values. PB is significantly better than CBZ for this outcome suggesting that PB is the most effective drug for delaying a first seizure.

### Generalized onset tonic clonic seizures

1790 (28%) patients were classified as having generalized onset tonic clonic seizures.

Results for time to treatment failure (Figure 4) indicate a significantly better outcome for individuals treated with VPA compared to CBZ, TPM and PB. There is a non-significant trend to suggest that VPA may be better than OXC and GBP but the confidence intervals are wide and include clinically important hazard ratios in both directions. There is a further non-significant trend in favour of VPA over LTG with a clinically non-significant lower limit of the confidence interval. There is insufficient evidence to differentiate between VPA and PHT with a wide confidence interval that contains clinically important results for both drugs. For 12 month remission (Figure 5), results suggest that VPA is significantly better than LTG. As the confidence intervals for the hazard ratio comparing each other drug with VPA each contain unity and clinically important values in both directions there is insufficient evidence to differentiate amongst the drugs. For the secondary outcome, time to first seizure (Figure 6), VPA is significantly better than LTG with non-significant trends to favour VPA over TPM, CBZ, PB and OXC. There is insufficient evidence to differentiate between VPA and PHT or GBP.

### Exchangeability

Although the time to event differed significantly according to year of recruitment, there was no systematic pattern of increasing hazard ratio over time for the any of the three outcomes. There is insufficient evidence to support the hypothesis that the effectiveness of CBZ increases over time and the assumption of exchangeability for this aspect appears reasonable. Further details are available on request from the first author.

### Consistency

For partial seizures there is evidence of statistical heterogeneity (qualitative) within the direct evidence for comparisons between CBZ and PB (time to treatment failure I^2 ^= 66% and time to 12 month remission I^2 ^= 67%), CBZ and VPA (time to first seizure I^2 ^= 78%). For generalized seizures there is evidence of statistical heterogeneity (qualitative with overlap of CIs) within the direct evidence for comparisons between PHT and VPA (time to treatment failure I^2 ^= 63%) and CBZ and PHT (time to 12 month remission I^2 ^= 73%).

Where comparisons are possible, the combined analysis (including all direct and indirect data) is consistent, in terms of hazard ratio and 95% confidence interval, with the direct evidence. However, for the comparison between VPA and TPM (partial seizures), the direct evidence (SANAD arm B with only 33 patients across the two drugs) suggests that TPM is significantly better than VPA (HR = 0.35; 95% CI = 0.15 to 0.80) for achieving a 12 month remission but there is insufficient evidence to differentiate between the two drugs in the combined analysis (HR = 1.00; 95% CI = 0.78 to 1.27). Further detail concerning the assessment of inconsistency can be made available from the authors.

For the comparison between CBZ and PB, the methodological quality of one included trial (Placencia 1993) was thought to be the main source of heterogeneity. As a sensitivity analysis, the combined multiple comparison analysis was undertaken excluding this particular trial. This reduced heterogeneity for the direct comparison but had minimal impact on interpretation and clinical conclusions for the combined multiple comparison analysis. The largest impact was seen for 12 month remission (partial subgroup) for which PB was less favoured in the sensitivity analysis [HR 1.22(0.90 to 1.64) in favour of CBZ for the sensitivity analysis compared to 1.01(0.77 to 1.31)].

## Discussion

In this paper we provide an up to date summary of multiple treatment comparisons from 20 randomised controlled trials (6418 patients) regarding the comparative effects of eight AEDs. The results represent the best available evidence about the comparative effects of these drugs, which will inform both clinical decision making and future research. Such results have not been available until now since no trial has, or is ever likely to compare all drugs directly.

The majority of the data (4628 (72%) patients) is for patients with partial onset seizures, and results favour OXC, CBZ, and LTG for the best combination of tolerability and seizure control, of which OXC and CBZ provide the best seizure control and LTG best tolerability resulting in a lower treatment failure rate.

A smaller number of patients classified as having generalized onset tonic-clonic seizures were recruited into the included randomised controlled trials (1790 patients (28%)). Some patients were experiencing other seizure types (absence or myoclonus) although data on these seizure types were not collected for the majority of trials during follow-up, hence seizure outcomes apply to generalized onset tonic clonic seizures only. The age distribution of patients reveals that approximately 30% were classified as having generalized seizures with onset over the age of 30 years, which is unlikely. For these patients it is likely that the randomizing clinician was unable to state with confidence that the seizures were partial in onset, and patients were therefore misclassified as generalized in onset. The group classified as having generalized onset seizures is therefore most likely to include some patients with partial onset seizures. Our results indicate that VPA has a lower treatment failure for all drugs except PHT. This is significant for PB, TPM, CBZ, and non significant for LTG, OXC and GBP. For time to 12 month remission results indicate that VPA is significantly superior to LTG and non-significantly superior to PB OXC and TPM, while PHT is non-significantly to VPA. These results do not refute current NICE guidelines that recommend VPA as a treatment of choice.

The results for patients with generalized onset tonic-clonic seizures are limited by the fact that fewer such patients have been recruited into randomised controlled trials, and that a significant number of patients classified as having generalized onset seizures may have been misclassified. Also, patients with an idiopathic generalized epilepsy may experience absence or myoclonic seizures. Other than in SANAD, these seizure types were not measured during follow-up, hence we do not know whether the 12 month remission rates here reflect an effect on generalized onset tonic-clonic seizures only, or upon all generalized seizure types. There is some uncontrolled evidence that PHT can exacerbate absence or myoclonus, and if true would have an important effect on measures of seizure remission were all generalized seizure types assessed during follow-up. Future trials must measure these shortfalls if they are to provide reliable information about relative treatment effects.

Previous meta-analyses have investigated individual pair-wise comparisons, which in isolation fall short of informing clinical decisions when there are a greater number of treatment options available. An advantage of the approach presented here is that it has allowed the full integration of evidence regarding eight AEDs and utilises data from the most recent and largest ever randomised controlled trial in epilepsy patients (SANAD). Analyses were performed using IPD from each trial. This approach allows the standardisation of outcome definition across trials. In particular we were able to assess the outcome for patients with either partial or generalized onset seizures and investigate the evidence to support prior clinical belief that for example CBZ is superior for partial onset seizures whilst VPA is superior for generalized onset seizures. A thorough examination of comparability of trial characteristics and an investigation into whether the effectiveness of CBZ had changed over time were also undertaken. These analyses would have been severely limited without IPD.

The key assumption made in the simultaneous analysis of multiple treatment comparisons is that the hazard ratio for one treatment compared with another would be the same across the entire set of trials included in the model [16] irrespective of whether those treatments were included in a particular trial. Caldwell et al [18] suggest that one way to question this assumption is to imagine all trials had compared the same treatments and to judge whether they are sufficiently similar to be combined in a meta-analysis. Each of the trials included in our analysis were assessed for eligibility into separate systematic reviews which all adopted the same inclusion criteria and review protocol and thus this decision had already been reached. We would encourage researchers undertaking multiple treatment comparisons to assess similarity of trials in this very way by developing a simple protocol describing trial eligibility criteria into the combined analysis as would be done in any properly conducted systematic review and meta-analysis for a single pair-wise comparison. Clinically, the trials are sufficiently similar to combine in a single analysis and justified further by the absence of a possible change in effect of CBZ over time and the lack of clinical belief of any contraindications to any of the anti-epileptic drugs in the included trials. The formulation of carbamazepine or valproate used is one potential source of clinical heterogeneity that could not be investigated due to lack of relevant data. Although the evidence for differing formulations' impact on outcome is limited, the controlled release formulation of carbamazepine or valproate may be associated with less treatment withdrawals due to adverse effects.

For the direct results there was evidence of heterogeneity amongst trial effects for a few comparisons for particular outcomes. Such heterogeneity may potentially bring about inconsistency between the direct and indirect evidence and could lead to difficulties with interpretation of the multiple treatment comparisons analysis. Consistency of the evidence is difficult to explore for this complex dataset. However, we generally found good consistency between the direct and combined multiple treatment comparisons results and found no systematic pattern of over or under estimation. This observation is in keeping with a previous study [17] that found the direction of discrepancy between direct and indirect estimates to be inconsistent. We did however find some evidence of inconsistency for the comparison between VPA and TPM (time to 12 month remission). However, only one trial with 33 patients provided direct evidence for this comparison. Further work is required to extend the multiple treatment comparisons IPD analysis presented to allow incorporation of heterogeneity which is likely to lead to increased confidence interval widths. This note of caution should be kept in mind when examining the presented results.

The simultaneous analysis of multiple treatment comparisons is increasingly more common in the medical literature with previous applications seen using aggregate data[42,43]. Caldwell et al [18] comment that a unified, coherent analysis can be achieved only by analysing the entire collection of relevant randomised controlled trials while respecting randomisation. They argue that rather than asking whether analyses comparing multiple treatments should be used routinely, it is more appropriate to ask whether they can be avoided. Comparing multiple treatments should be considered as the bedrock for decisions when several treatments are available [18]. The use of IPD, with its associated benefits, can only strengthen such decision-making.

## Conclusion

### Implications for practice

For patients with partial onset seizures, results favour OXC, CBZ and LTG as drugs of choice. It will be noted that all these drugs have their primary mode of action as voltage dependent and use dependent blockers of Na^+ ^channels. The data presented here support the current guidelines recommending the use of VPA as drug of first choice for generalized onset tonic-clonic seizures. However the potential teratogenicity of VPA should be considered for women of child bearing age.

### Implications for research

The paucity of data for patients with generalized onset tonic clonic seizures highlights the need for trials into which such individuals are recruited. The problems with classification that are highlighted should be addressed in future trials in which systems should be used to check seizure and epilepsy classification on the one hand, whilst allowing clinicians to express their uncertainty about classification on the other. These analyses will be continually updated as new data become available.

## Competing interests

Tudur Smith C and Williamson PR have reported no conflicts of interest. Marson AG has received speaker fees and reimbursement for attending conferences from Janssen-Cilag, Glaxo SmithKline, Novartis, Pfizer and Sanofi Synthelabo and research funding from Pfizer. Chadwick DW has received consultancy fees, speakers fees and reimbursement for attending conferences from Janssen-Cilag, Glaxo SmithKline, Novartis, Pfizer and Sanofi Synthelabo

## Authors' contributions

Catrin Tudur Smith, participated in developing ideas and methods, undertook all analyses, produced graphical presentation of results and participated in interpretation of results and writing of the manuscript. Anthony Marson obtained original IPD and liaised with trialists, participated in developing ideas, provided clinical support and advice, participated in presentation and interpretation of results and writing of the manuscript. David Chadwick helped to obtain original IPD, provided clinical support and advice, participated in the interpretation of results and writing of the manuscript. Paula Williamson, proposed a multiple treatment comparisons analysis, participated in developing ideas and methods, provided statistical support and advice for analyses, presentation and interpretation of results, and participated in writing the manuscript.

## Appendix 1. Model for the analysis of multiple treatment comparisons

For the simultaneous analysis of multiple comparisons between eight AEDs, a Cox proportional hazards model stratified by trial assuming fixed treatment effects was fitted, where the hazard for the *ith *patient (*i *= 1,..., *n*_*j*_) in the *jth *trial (*j *= 1,..., *J*) is given by

*λ*_*ij *_= *λ*_0*j*_(*t*)exp(*β*_1_*x*_1*ij *_+ *β*_2_*x*_2*ij *_+ *β*_3_*x*_3*ij *_+ *β*_4_*x*_4*ij *_+ *β*_5_*x*_5*ij*_)

where *x*_*k*_'s are treatment indicator variables and *β*_*k *_are regression coefficients.

For comparisons with the baseline drug (CBZ in this example), the hazard ratio is given by exp⁡(β^k)
 MathType@MTEF@5@5@+=feaafiart1ev1aaatCvAUfKttLearuWrP9MDH5MBPbIqV92AaeXatLxBI9gBaebbnrfifHhDYfgasaacPC6xNi=xH8viVGI8Gi=hEeeu0xXdbba9frFj0xb9qqpG0dXdb9aspeI8k8fiI+fsY=rqGqVepae9pg0db9vqaiVgFr0xfr=xfr=xc9adbaqaaeGacaGaaiaabeqaaeqabiWaaaGcbaGagiyzauMaeiiEaGNaeiiCaaNaeiikaGccciGaf8NSdiMbaKaadaWgaaWcbaGaem4AaSgabeaakiabcMcaPaaa@3506@ with 95% confidence interval exp⁡(β^k±1.96∗SE(β^k))
 MathType@MTEF@5@5@+=feaafiart1ev1aaatCvAUfKttLearuWrP9MDH5MBPbIqV92AaeXatLxBI9gBaebbnrfifHhDYfgasaacPC6xNi=xH8viVGI8Gi=hEeeu0xXdbba9frFj0xb9qqpG0dXdb9aspeI8k8fiI+fsY=rqGqVepae9pg0db9vqaiVgFr0xfr=xfr=xc9adbaqaaeGacaGaaiaabeqaaeqabiWaaaGcbaGagiyzauMaeiiEaGNaeiiCaaNaeiikaGccciGaf8NSdiMbaKaadaWgaaWcbaGaem4AaSgabeaakiabgglaXkabigdaXiabc6caUiabiMda5iabiAda2iabgEHiQiabdofatjabdweafjabcIcaOiqb=j7aIzaajaWaaSbaaSqaaiabdUgaRbqabaGccqGGPaqkcqGGPaqkaaa@42E6@ for k = 1,..., 5.

For other pair wise comparisons between drugs represented by dummy variables *x*_*kij *_and *x*_*mij *_the hazard ratio is given by exp⁡[(β^k−β^m)]
 MathType@MTEF@5@5@+=feaafiart1ev1aaatCvAUfKttLearuWrP9MDH5MBPbIqV92AaeXatLxBI9gBaebbnrfifHhDYfgasaacPC6xNi=xH8viVGI8Gi=hEeeu0xXdbba9frFj0xb9qqpG0dXdb9aspeI8k8fiI+fsY=rqGqVepae9pg0db9vqaiVgFr0xfr=xfr=xc9adbaqaaeGacaGaaiaabeqaaeqabiWaaaGcbaGagiyzauMaeiiEaGNaeiiCaaNaei4waSLaeiikaGccciGaf8NSdiMbaKaadaWgaaWcbaGaem4AaSgabeaakiabgkHiTiqb=j7aIzaajaWaaSbaaSqaaiabd2gaTbqabaGccqGGPaqkcqGGDbqxaaa@3BB8@ with standard error

SE(β^k−β^m)=SE(β^k)2+SE(β^m)2−2∗cov⁡(β^k,β^m)
 MathType@MTEF@5@5@+=feaafiart1ev1aaatCvAUfKttLearuWrP9MDH5MBPbIqV92AaeXatLxBI9gBaebbnrfifHhDYfgasaacPC6xNi=xI8qiVKYPFjYdHaVhbbf9v8qqaqFr0xc9vqFj0dXdbba91qpepeI8k8fiI+fsY=rqGqVepae9pg0db9vqaiVgFr0xfr=xfr=xc9adbaqaaeGacaGaaiaabeqaaeqabiWaaaGcbaGaem4uamLaemyrauKaeiikaGccciGaf8NSdiMbaKaadaWgaaWcbaGaem4AaSgabeaakiabgkHiTiqb=j7aIzaajaWaaSbaaSqaaiabd2gaTbqabaGccqGGPaqkcqGH9aqpdaGcaaqaaiabdofatjabdweafjabcIcaOiqb=j7aIzaajaWaaSbaaSqaaiabdUgaRbqabaGccqGGPaqkdaahaaWcbeqaaiabikdaYaaakiabgUcaRiabdofatjabdweafjabcIcaOiqb=j7aIzaajaWaaSbaaSqaaiabd2gaTbqabaGccqGGPaqkdaahaaWcbeqaaiabikdaYaaakiabgkHiTiabikdaYiabgEHiQiGbcogaJjabc+gaVjabcAha2jabcIcaOiqb=j7aIzaajaWaaSbaaSqaaiabdUgaRbqabaGccqGGSaalcuWFYoGygaqcamaaBaaaleaacqWGTbqBaeqaaOGaeiykaKcaleqaaaaa@5A67@

The model can be adjusted for the effect of covariate and treatment-by-covariate interaction terms by including relevant dummy variables in the linear predictor.

## Supplementary Material

Additional file 1 Figure X. Supplementary figures with full details for all outcomes Click here for file

Additional file 2 Table 1. Characteristics of trials ad patients included in simultaneous analysis of multiple treatment comparisons Click here for file
