# Supplementary material for: Multiple treatment comparisons in epilepsy monotherapy trials
Source: Trials. 2007 Nov 5;8:34. doi: 10.1186/1745-6215-8-34 (PMC2194733; doi:10.1186/1745-6215-8-34)
Supplement: Additional file 1 — Figure X. Supplementary figures with full details for all outcomes [file 1745-6215-8-34-S1.doc]

**Time to withdrawal for partial onset seizures**

*0.5*

*1*

*2*

*5*

*phb*

*1.29 (0.98, 1.69)*

*pht*

*1.07 (0.80, 1.43)*

*phb*

*1.38 (0.99, 1.91)*

*gbp*

*1.03 (0.84, 1.26)*

*pht*

*1.10 (0.82, 1.47)*

*phb*

*1.42 (1.02, 1.96)*

*tpm*

*1.12 (0.85, 1.48)*

*gbp*

*1.16 (0.88, 1.52)*

*pht*

*1.23 (0.93, 1.64)*

*phb*

*1.59 (1.15, 2.19)*

*vps*

*1.00 (0.82, 1.24)*

*tpm*

*1.13 (0.93, 1.37)*

*gbp*

*1.16 (0.96, 1.41)*

*pht*

*1.24 (0.98, 1.57)*

*phb*

*1.60 (1.22, 2.10)*

*cbz*

*1.14 (0.90, 1.45)*

*vps*

*1.15 (0.84, 1.56)*

*tpm*

*1.29 (0.99, 1.67)*

*gbp*

*1.32 (1.02, 1.72)*

*pht*

*1.41 (1.05, 1.90)*

*phb*

*1.82 (1.29, 2.57)*

*oxc*

*1.26 (0.97, 1.63)*

*cbz*

*1.43 (1.20, 1.71)*

*vps*

*1.44 (1.11, 1.87)*

*tpm*

*1.62 (1.31, 1.99)*

*gbp*

*1.66 (1.35, 2.05)*

*pht*

*1.77 (1.34, 2.35)*

*phb*

*2.29 (1.67, 3.14)*

**LTG**

**OXC**

**CBZ**

**VPS**

**TPM**

**PHT**

**GBP**

**AED 1 AED 2**

**CBZ**

**VPS**

**TPM**

**GBP**

**PHT**

*0.5*

*1*

*2*

*5*

HR>1 Baseline AED better

HR<1 Baseline AED worse

HR<1 AED 1 worse

HR>1 AED 1 better

**Time to withdrawal for generalised onset seizures**

*0.1*

*0.2*

*0.5*

*1*

*2*

*5*

*10*

*100*

*phb*

*1.05 (0.59, 1.88)*

*tpm*

*1.09 (0.15, 7.99)*

*phb*

*1.15 (0.15, 8.76)*

*gbp*

*1.06 (0.14, 8.12)*

*tpm*

*1.16 (0.63, 2.15)*

*phb*

*1.22 (0.59, 2.52)*

*oxc*

*1.04 (0.58, 1.86)*

*gbp*

*1.10 (0.15, 7.95)*

*tpm*

*1.20 (0.83, 1.74)*

*phb*

*1.26 (0.74, 2.15)*

*cbz*

*1.11 (0.79, 1.57)*

*oxc*

*1.15 (0.63, 2.10)*

*gbp*

*1.22 (0.17, 8.87)*

*tpm*

*1.34 (0.98, 1.82)*

*phb*

*1.41 (0.80, 2.47)*

*ltg*

*1.26 (0.82, 1.94)*

*cbz*

*1.40 (0.93, 2.11)*

*oxc*

*1.45 (0.79, 2.67)*

*gbp*

*1.54 (0.21, 11.33)*

*tpm*

*1.68 (1.07, 2.64)*

*phb*

*1.77 (0.99, 3.17)*

*pht*

*1.03 (0.71, 1.51)*

*ltg*

*1.30 (0.97, 1.75)*

*cbz*

*1.45 (1.07, 1.96)*

*oxc*

*1.50 (0.84, 2.68)*

*gbp*

*1.59 (0.22, 11.50)*

*tpm*

*1.74 (1.28, 2.36)*

*phb*

*1.83 (1.07, 3.13)*

HR>1 AED 1 better

HR<1 AED 1 worse

**VPS**

**PHT**

**LTG**

**CBZ**

**OXC**

**GBP**

**TPM**

**AED 1 AED 2**

**Time to 12 month remission for partial onset seizures**

*0.5*

*1*

*2*

*gbp*

*1.15 (0.90, 1.47)*

*vps*

*1.00 (0.79, 1.28)*

*gbp*

*1.16 (0.95, 1.41)*

*tpm*

*1.04 (0.80, 1.35)*

*vps*

*1.04 (0.83, 1.31)*

*gbp*

*1.20 (0.93, 1.57)*

*pht*

*1.00 (0.78, 1.29)*

*tpm*

*1.04 (0.86, 1.26)*

*vps*

*1.04 (0.83, 1.32)*

*gbp*

*1.21 (0.99, 1.46)*

*ltg*

*1.14 (0.84, 1.55)*

*pht*

*1.14 (0.87, 1.50)*

*tpm*

*1.19 (0.87, 1.62)*

*vps*

*1.19 (0.89, 1.59)*

*gbp*

*1.37 (1.00, 1.88)*

*phb*

*1.01 (0.74, 1.38)*

*ltg*

*1.15 (0.92, 1.43)*

*pht*

*1.15 (0.90, 1.47)*

*tpm*

*1.19 (0.95, 1.50)*

*vps*

*1.20 (0.93, 1.54)*

*gbp*

*1.38 (1.10, 1.74)*

*oxc*

*1.00 (0.82, 1.22)*

*phb*

*1.01 (0.77, 1.31)*

*ltg*

*1.15 (0.96, 1.37)*

*pht*

*1.15 (0.94, 1.41)*

*tpm*

*1.19 (0.99, 1.43)*

*vps*

*1.20 (1.01, 1.42)*

*gbp*

*1.38 (1.15, 1.67)*

**CBZ**

**OXC**

**PHB**

**LTG**

**PHT**

**TPM**

**VPS**

**AED 1 AED 2**

HR>1 AED 1 better

HR<1 AED 1 worse

**Time to 12 month remission for generalised onset seizures**

*0.2*

*0.5*

*1*

*2*

*5*

*10*

*100*

*ltg*

*1.10 (0.72, 1.68)*

*phb*

*1.16 (0.70, 1.93)*

*ltg*

*1.28 (0.80, 2.04)*

*oxc*

*1.01 (0.64, 1.61)*

*phb*

*1.18 (0.77, 1.79)*

*ltg*

*1.29 (1.01, 1.67)*

*tpm*

*1.09 (0.86, 1.37)*

*oxc*

*1.10 (0.73, 1.67)*

*phb*

*1.28 (0.89, 1.84)*

*ltg*

*1.41 (1.10, 1.80)*

*vps*

*1.00 (0.82, 1.23)*

*tpm*

*1.09 (0.81, 1.47)*

*oxc*

*1.11 (0.72, 1.70)*

*phb*

*1.29 (0.89, 1.85)*

*ltg*

*1.41 (1.05, 1.92)*

*cbz*

*1.08 (0.83, 1.41)*

*vps*

*1.09 (0.85, 1.39)*

*tpm*

*1.18 (0.85, 1.63)*

*oxc*

*1.20 (0.79, 1.82)*

*phb*

*1.39 (0.95, 2.03)*

*ltg*

*1.53 (1.10, 2.13)*

*pht*

*3.56 (0.49, 25.71)*

*cbz*

*3.85 (0.54, 27.68)*

*vps*

*3.87 (0.54, 27.75)*

*tpm*

*4.21 (0.58, 30.47)*

*oxc*

*4.27 (0.58, 31.57)*

*phb*

*4.95 (0.67, 36.34)*

*ltg*

*5.45 (0.75, 39.51)*

**AED 1 AED 2**

HR<1 AED 1 worse

HR>1 AED 1 better

**GBP**

**PHT**

**CBZ**

**VPS**

**TPM**

**OXC**

**PHB**

**Time to first seizure for partial onset seizures**

*0.5*

*1*

*2*

*5*

*gbp*

*1.05 (0.89, 1.23)*

*ltg*

*1.05 (0.88, 1.26)*

*gbp*

*1.10 (0.90, 1.36)*

*vps*

*1.18 (0.97, 1.42)*

*ltg*

*1.24 (1.01, 1.51)*

*gbp*

*1.30 (1.04, 1.61)*

*pht*

*1.04 (0.84, 1.30)*

*vps*

*1.22 (1.00, 1.50)*

*ltg*

*1.29 (1.09, 1.52)*

*gbp*

*1.35 (1.14, 1.61)*

*tpm*

*1.00 (0.85, 1.18)*

*pht*

*1.04 (0.88, 1.24)*

*vps*

*1.23 (1.06, 1.41)*

*ltg*

*1.29 (1.13, 1.48)*

*gbp*

*1.35 (1.15, 1.59)*

*cbz*

*1.01 (0.84, 1.21)*

*tpm*

*1.01 (0.82, 1.24)*

*pht*

*1.05 (0.86, 1.29)*

*vps*

*1.23 (0.99, 1.53)*

*ltg*

*1.30 (1.07, 1.57)*

*gbp*

*1.36 (1.11, 1.67)*

*oxc*

*1.30 (0.99, 1.70)*

*cbz*

*1.30 (1.04, 1.63)*

*tpm*

*1.31 (1.00, 1.71)*

*pht*

*1.36 (1.08, 1.71)*

*vps*

*1.60 (1.25, 2.05)*

*ltg*

*1.68 (1.30, 2.17)*

*gbp*

*1.77 (1.35, 2.31)*

**Baseline AED**

**PHB**

**OXC**

**CBZ**

**TPM**

**PHT**

**VPS**

**LTG**

HR>1 Baseline AED better

HR<1 Baseline AED worse

**Time to first seizure for generalised onset seizures**

*0.1*

*0.2*

*0.5*

*1*

*2*

*5*

*10*

*ltg*

*1.11 (0.74, 1.67)*

*oxc*

*1.03 (0.65, 1.65)*

*ltg*

*1.15 (0.81, 1.64)*

*phb*

*1.06 (0.76, 1.47)*

*oxc*

*1.09 (0.74, 1.62)*

*ltg*

*1.22 (0.97, 1.53)*

*cbz*

*1.01 (0.77, 1.33)*

*phb*

*1.07 (0.73, 1.57)*

*oxc*

*1.11 (0.72, 1.69)*

*ltg*

*1.23 (0.97, 1.56)*

*tpm*

*1.07 (0.15, 7.68)*

*cbz*

*1.08 (0.15, 7.72)*

*phb*

*1.15 (0.16, 8.33)*

*oxc*

*1.19 (0.16, 8.67)*

*ltg*

*1.32 (0.19, 9.41)*

*gbp*

*1.11 (0.16, 7.90)*

*tpm*

*1.19 (0.94, 1.51)*

*cbz*

*1.21 (0.99, 1.47)*

*phb*

*1.28 (0.92, 1.77)*

*oxc*

*1.32 (0.90, 1.94)*

*ltg*

*1.47 (1.20, 1.80)*

*vps*

*1.03 (0.81, 1.30)*

*gbp*

*1.14 (0.16, 8.17)*

*tpm*

*1.23 (0.90, 1.66)*

*cbz*

*1.24 (0.96, 1.59)*

*phb*

*1.31 (0.93, 1.86)*

*oxc*

*1.35 (0.91, 2.02)*

*ltg*

*1.51 (1.15, 1.98)*

HR>1 Baseline AED better

HR<1 Baseline AED worse

**Baseline AED**

**PHT**

**VPS**

**GBP**

**TPM**

**CBZ**

**PHB**

**OXC**
