# Supplementary material for: Multiple treatment comparisons in epilepsy monotherapy trials
Source: Trials. 2007 Nov 5;8:34. doi: 10.1186/1745-6215-8-34 (PMC2194733; doi:10.1186/1745-6215-8-34)
Supplement: Additional file 2 — Table 1. Characteristics of trials ad patients included in simultaneous analysis of multiple treatment comparisons [file 1745-6215-8-34-S2.doc]

#### Table 1. Characteristics of trials ad patients included in simultaneous analysis of multiple treatment comparisons

| **Trial** | **Recruitment period** | AED (number patients)^1^ | | | | | | | | **Previous**  **seizures** | **Age years** Mean (SD) range | **Partial**  **Epilepsy**  (%) | **Number of seizures^5^**  median  (25^th^,75^th^ centile) | **Previous AED** | **Blinding** |
| --- | --- | --- | --- | --- | --- | --- | --- | --- | --- | --- | --- | --- | --- | --- | --- |
|  |  | **CBZ** | **PB** | **PHT** | **VPA** | **LTG** | **OXC** | **GBP** | **TPM** |  |  |  |  |  |  |
| Heller  1995 [27] | Feb 1981 to  Aug 1987 | **61** | **58** | 63 | **61** | - | - | - | - | ≥ 2 GTC seizures or PS ± secondary generalisation in preceding year | 32 (14.8)  13-77 | 42 | 2(2,6) | Untreated | Open |
| De Silva  1996 [28] | Dec 1980 to  Sep 1987 | **54** | **10** | **54** | **49** | - | - | - | - | ≥ 2 GTC seizures or PS ± secondary generalisation in preceding year | 9.9 (3.6)  3-16 | 53 | 3(2,10) | Untreated | Open |
| Mattson 1985 [29] | June 1978 to  July 1983 | **154** | **155** | **165** | - | - | - | - | - | Simple or complex partial or secondarily GTC seizures | 41 (15.5)  18-82 | 100 | 1(1,3) | Untreated or under treated | Double blind |
| Mattson 1992 [30] | Feb 1985 to  Feb 1990 | **231** | - | - | **239** | - | - | - | - | Complex partial, secondarily generalised GTC seizures, or both | 47.3 (16.2)  18-83 | 100 | 12(4,96) | Untreated or under treated | Double blind |
| Richens 1994 [31] | May 1982 to  April 1987 | **146** | - | - | **144** | - | - | - | - | ≥ 2 GTC seizures or PS ± secondary generalisation in previous 6m | 33.3 (15.0)  16-79 | 49 | 4(2,10) | Untreated (98%) | Open |
| Verity  1995 [32] | Nov 1985 to  Dec 1987 | **127** | - | - | **119** | - | - | - | - | ≥ 2 GTC seizures or PS ± secondary generalisation in previous 6m | 10.0 (2.9)  5-16 | 42 | 3(2,6) | Untreated or seizures recurred | Open |
| Brodie 1995a^2^ [33]^Φ^ | April 1989 to  Dec 1992 | **66** | - | - | - | **70** | - | - | - | ≥ 2 PS or GTC seizures in previous 6 months | 34 (15.8)  13-71 | 59 | 4(2,17) | Untreated | Double blind |
| Brodie 1995b^2^ [33] ^Φ^ | April 1989 to  Dec 1992 | **63** | - | - | - | **61** | - | - | - | ≥ 2 PS or GTC seizures in previous 6 months | 30 (14.1)  14-81 | 46 | 3(2,7.5) | Untreated | Double blind |
| Reunanen 1996^2,3^ [34] | March 1990 to  March 1993 | **120** | - | - | - | **229** | - | - | - | ≥ 2 PS ± GTC seizures in previous 6 months | 32.1 (14.2)  12-72 | 65 | 3(2,9) | Untreated or recurrent epilepsy | Open |
| Ramsay 1992^2^ [35] | Oct 1979 to  July 1982 | - | - | **50** | **86** | - | - | - | - | Newly diagnosed primary GTC seizures with ≥ 2 seizures within 14 days | 21 (14.2)  3-64 | 0 | NA | Untreated, or seizure free without AED | Open |
| Craig  1994^3^ [36] | Jan 1990 to  Dec 1991 | - | - | **71** | **76** | - | - | - | - | ≥1 GTC seizure or ≥2 PS | 78.1 (7)  61-95 | 50 | 3(2,6) | Unclear | Open |
| Turnbull 1985 [37] | Oct 1978 to  Dec 1980 | - | - | **70** | **70** | - | - | - | - | ≥2 GTC and PS in previous 3 years and last seizure within 3 months | 35.2 (16.1)  14-70 | 45 | 2(1,5) | Untreated | Open |
| Placencia 1993 [38] | Oct 1986 to  Aug 1987 | **95** | **97** | - | - | - | - | - | - | ≥2 afebrile seizures in previous year | 29 (17.6)  2-68 | 70 | 2(1,4) | Untreated or previously treated | Open |
| Brodie 1999^2^ [39] | Dec 1994 to  April 1997 | **48** | - | - | - | **102** | - | - | - | ≥ 2 seizures of any type in previous year | 76.9 (6)  65-94 | 70 | 3(2,10) | Untreated | Double blind |
| Bill  1997 [40] | July 1991 to  Jan 1994 | - | - | **144** | - | - | **143** | - | - | ≥2 PS or GTC seizures in preceding 6 months | 26.8 (10.7)  15-91 | 64 | 3(2,8) | Untreated | Double blind |
| Guerreiro 1997 [41] | July 1991 to  Nov 1993 | - | - | **96** | - | - | **97** | - | - | ≥2 PS or GTC seizures in preceding 6 months | 10.5 (3.1)  5-17 | 79 | 2(2,4) | Untreated | Double blind |
| Pal  1998^3^ [42] | Aug 1995 to  Feb 1997 | - | **47** | **47** | - | - | - | - | - | ≥2 unprovoked seizures in preceding year | 11.4 (5)  2-18 | 64 | NA | Untreated in 3 months | Open |
| Barrera 2001^2,4^ [43] | Aug 1995 to  Feb 1998 | **202** | - | - | - | **420** | - | - | - | ≥ 2 seizures in previous 6 months with ≥ 1 PS or secondarily GTC seizure in 3 months | 27.2 (21.4)  2-83 | 99 | 3(2,13) | Newly diagnosed or currently untreated | Open |
| Marson et al  2006 [6] (SANAD A) | Jan 1999 to  Aug 2004 | **364** | **-** | **-** | **-** | **371** | **202** | **366** | **369** | ≥ 2 clinically definite unprovoked epileptic seizures in the previous year | 38.4 (18.4)  5-86 | 98 | 4 (2,11) | Newly diagnosed and untreated, treated with ineffective monotherapy, relapse after remission of epilepsy | Open |
| Marson et al 2006 [7] (SANAD B) | Jan 1999 to  Aug 2004 | **-** | - | - | **234** | **232** | - | - | **233** | ≥ 2 clinically definite unprovoked epileptic seizures in the previous year | 22.5 (14.1)  5-77 | 10 | 3 (2, 19) | Newly diagnosed and untreated, treated with ineffective monotherapy, relapse after remission of epilepsy | Open |
|  | **TOTAL** | **1731** | **367** | **760** | **1078** | **1485** | **442** | **366** | **602** |  |  |  |  |  |  |

^1^ Number of patients with data available for at least one outcome. Number may vary according to analysis if outcome or epilepsy type data missing.

^2^ Data unavailable for time to 12 month remission

^3^ Data unavailable for time to treatment failure

^4^ Data unavailable for time to first seizure

^5^ Number of seizures in 6 months before randomisation

^Φ^ Reference 33 reports results from two independent trials referred to as Brodie 1995a and Brodie 1995b.

**SD**: Standard Deviation**, CBZ**: Carbamazepine, **VPA**: Sodium Valproate, **PHT**: Phenytoin, **PB**: Phenobarbitone, **LTG**: Lamotrigine, **OXC**: Oxcarbazepine, **GBP**: Gabapentine, **TPM**: Topirimate

PS: Partial Seizures

GTC: Generalised Tonic Clonic

AED: Antiepileptic Drug

NA: Data not available
